# Odoriferous

**Published:** 1870-07

**Authors:** 


### ODORIFEROUS.

Especially in warm weather, there is an odor attached to some persons, particularly in the feet, or under the arms. Get a few ounces of hartshorn water at a drug-store, and after washing the feet and armpits, at bedtime, with soap and warm water, wipe dry, and with the hand or fingers apply a teaspoonful or two to the arm-pits and feet, especially between the toes. By morning, the odor of the hartshorn will have been dissipated from the person; after a few applications, its use can be discontinued. The perspiration, moist or dry, is an acid, and is antagonized by the hartshorn, which is a strong alkali.

### POISONOUS BITES.

The bites and stings of all animals and insects become poisonous from the acid nature of the liquid which is injected into the circulation at the instant of the bite or sting, and if strong spirits of hartshorn could be instantly applied the poison would be instantaneously antagonized. Even in the case of a mad-dog bite, if the hartshorn could be promptly introduced into the system by injecting it under the skin, as is said to be done in India, there would be additional certainty of exemption from injury. After all bites and stings, a bag saturated with hartshorn, and kept so for several hours, should be applied.

